# Error propagation in constraint‐based modeling of Chinese hamster ovary cells

**DOI:** 10.1002/biot.202000320

**Published:** 2021-01-06

**Authors:** Diana Széliová, Dmytro Iurashev, David E Ruckerbauer, Gunda Koellensperger, Nicole Borth, Michael Melcher, Jürgen Zanghellini

**Affiliations:** ^1^ Department of Biotechnology University of Natural Resources and Life Sciences Vienna Austria; ^2^ acib – Austrian Centre of Industrial Biotechnology Vienna Austria; ^3^ Department of Analytical Chemistry University of Vienna Vienna Austria; ^4^ Institute of Statistics University of Natural Resources and Life Sciences Vienna Austria; ^5^ Present address: Währinger Str. 38 Vienna EU 1090 Austria

**Keywords:** Chinese hamster ovary cells, error propagation, exchange rates, genome‐scale metabolic modeling, flux balance analysis

## Abstract

Chinese hamster ovary (CHO) cells are the most popular mammalian cell factories for the production of glycosylated biopharmaceuticals. To further increase titer and productivity and ensure product quality, rational system‐level engineering strategies based on constraint‐based metabolic modeling, such as flux balance analysis (FBA), have gained strong interest. However, the quality of FBA predictions depends on the accuracy of the experimental input data, especially on the exchange rates of extracellular metabolites. Yet, it is not standard practice to devote sufficient attention to the accurate determination of these rates. In this work, we investigated to what degree the sampling frequency during a batch culture and the measurement errors of metabolite concentrations influence the accuracy of the calculated exchange rates and further, how this error then propagates into FBA predictions of growth rates. We determined that accurate measurements of essential amino acids with low uptake rates are crucial for the accuracy of FBA predictions, followed by a sufficient number of analyzed time points. We observed that the measured difference in growth rates of two cell lines can only be reliably predicted when both high measurement accuracy and sampling frequency are ensured.

## Graphical abstract

Chinese hamster ovary (CHO) cells are the most important hosts for the production of complex protein biopharmaceuticals and their performance could be improved by rational engineering approaches predicted by metabolic modeling. The influence and importance of experimental errors and sampling frequency on the predictive qualities of flux balance analysis in cell culture are analysed here.

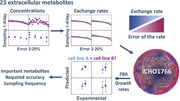

**Abbreviations**
AA: amino acid
CHO: Chinese hamster ovary
FBA: flux balance analysis
GSMM: genome‐scale metabolic model
RSD: relative standard deviation
RSE: relative standard error
SE: standard error

## 1 INTRODUCTION

Chinese hamster ovary (CHO) cells are the primary host for the production of biopharmaceuticals, particularly monoclonal antibodies and other complex therapeutic proteins.^[^ ^1^ ^]^ Their main advantages include the ability to perform human‐like post‐translational modifications, in particular glycosylation, suspension growth in serum‐free chemically defined media and a low risk of viral infections, which makes them a safe host for the production of human therapeutic proteins.^[^ ^2^, ^3^ ^]^ However, the development of new producer cell lines is a time‐consuming, costly, and laborious process, based mostly on laboratory evolution and high‐throughput screening of thousands of clones. It often takes several months to obtain a high producer and this trial‐and‐error process must be repeated for each new product.^[^ ^4^ ^]^ Furthermore, it is not clear which factors limit product formation.^[^ ^5^ ^]^

As a result of the success of metabolic modeling in designing microbial cell factories,^[^ ^6^ ^]^ interest has grown in applying these methods to CHO.^[^ ^7^ ^]^ However, applications of modeling to CHO cells remain limited as key (bioinformatic) resources became available only recently. The publication of CHO's genome sequence^[^ ^8^ ^]^ enabled the in silico reconstruction of its metabolism.^[^ ^9^ ^]^ Along with recent updates,^[^ ^10^, ^11^ ^]^ these genome‐scale metabolic models (GSMMs) sit at the heart of constraint‐based modeling approaches that allow to computationally connect genotype and phenotype.^[^ ^12^ ^]^ In fact, such a GSMM coupled with a model of the secretory pathway^[^ ^13^ ^]^ validated gene knockouts that led to increased productivity and growth.^[^ ^14^ ^]^ Hence, genome‐scale modeling has great potential to improve CHO cell line development by identifying bottlenecks in productivity and designing engineering strategies to resolve these.

Constraint‐based modeling, such as FBA,^[^ ^15^ ^]^ is a common approach for the analysis of GSMMs.^[^ ^16^ ^]^ The accuracy of FBA predictions depends on the correct reconstruction of the metabolic pathways, the biomass composition and the exchange rates of extracellular metabolites which are used as constraints.^[^ ^15^ ^]^ Previously we showed that the quality of growth rate predictions by FBA depends on the accuracy of the measured exchange rates.^[^ ^17^ ^]^ While simple organisms such as bacteria or yeast can grow on a single carbon source (thereby simplifying the analysis), mammalian cells grow in complex media containing numerous essential metabolites and secrete several byproducts. Typically, more than 20 exchange rates have to be measured in order to perform FBA, which makes the analysis much more challenging than for simpler organisms on minimal media. Despite the importance and the difficulty of determining accurate exchange rates for mammalian cells, little attention has been paid to this topic so far.

To determine exchange rates accurately, it is necessary to measure the metabolite concentration throughout the cultivation at a sufficient sampling frequency. In the literature, the concentrations of extracellular metabolites are commonly measured once per day, typically resulting in four to six time points in total.^[^ ^18^, ^19^, ^20^, ^21^, ^22^, ^23^, ^24^, ^25^ ^]^ However, this might not be enough to obtain accurate exchange rates.

Here we investigated the impact of sampling frequency and the error of metabolite concentration measurements on the calculation of exchange rates and subsequently on FBA predictions of growth rate. We determined which exchange rates have the biggest impact on FBA and what accuracy and sampling frequency is required to detect a given difference between two cell lines.

## 2 METHODS

The simulations, statistical analysis and visualization were done in R version 4.0.3.^[^ ^26^ ^]^ FBA^[^ ^16^ ^]^ was performed in python 3.7.4 using package COBRApy^[^ ^27^ ^]^ with Gurobi solver. All data and scripts are available at https://doi.org/10.17632/5vn5m33wpr.1.

### 2.1 Reference FBA

To generate a reference state, FBA was performed with the experimental data and GSMMs from Széliová et al.^[^ ^17^ ^]^ for 11 out of thirteen available datasets (two datasets (GScd4‐8mMCD and DXepo‐0mMCD) were omitted from the analysis due to very inaccurate growth rate predictions). The experimentally measured exchange rates of glucose, amino acids (AAs), lactate and ammonium were used as constraints (see Table A1 for two example cell lines – K1par‐8mMAP and HYher‐8mMCD; see link above for the full dataset). Cysteine and tryptophan uptakes were left unconstrained due to the lack of quantitative experimental data. Biomass production was maximized (reaction “R_biomass_specific”). Then, growth rate was fixed to the predicted value and the uptakes of cysteine and tryptophan were minimized to obtain estimates for their uptake rates. Lactate secretion was left unconstrained for the cell line DGpar‐8mMCD as in the original paper (otherwise the predicted cysteine uptake rate was unrealistically high—higher than the glucose uptake rate). The predicted growth rates were used as reference states in the subsequent analysis.

### 2.2 Simulation of concentration profiles

The experimental exchange rates from Széliová et al.,^[^ ^17^ ^]^ the predicted growth rates and the uptake rates of cysteine and tryptophan from the reference FBA (see Section 2.1) were used to simulate concentration profiles using Equation (1),

(1) $$\left[\hat{i}\right]={\left[\hat{i}\right]}_{0}+\frac{{\hat{q}}_{i}{\hat{\text{BM}}}_{0}}{\hat{\mu }}\left(e^{\hat{\mu }t}-1\right),$$

where $\left[\hat{i}\right]$ is an ideal concentration of metabolite i during exponential phase, ${\left[\hat{i}\right]}_{0}$ is the initial concentration of metabolite i in the medium, ${\hat{q}}_{i}$ is the reference exchange rate, $\hat{\mu }$ is the growth rate, t is time of cultivation and ${\hat{\text{BM}}}_{0}$ is the initial amount of biomass, calculated from an initial cell concentration ($1.6\times 10^{5}$ viable cells per mL) and the experimentally measured dry mass (K1par‐8mMAP: 252.3 pg per cell, HYher‐8mMCD: 279 pg per cell, see link above for the full dataset). The values for ${\left[\hat{i}\right]}_{0}$, ${\hat{q}}_{i}$, cell dry mass and initial cell concentration were taken from Széliová et al.^[^ ^17^ ^]^ (Table A1). $\hat{\mu }$ is the growth rate predicted by the reference FBA. The time of cultivation was 90 h (the mean length of exponential phases of the two example cell lines). Samples were generated at regular intervals of 6, 12, or 24 h or—to resemble typical working shifts—at irregular intervals with four samples per day—every 4 h with a 12 h gap or every 2.5 h with a 16.5 h gap (the gap was positioned either in the beginning of each day or after the first four ”dense” sampling time points, e.g., hours 0, 12, 16, 20, 24, 36 etc. or 0, 4, 8, 12, 24, 30 etc.).

To simulate experimental data, normally distributed noise was added to the ideal concentrations

(2) $$\left[i\right]=\left[\hat{i}\right]+\varepsilon_{c}\;\text{with}\;\varepsilon_{c}∼N\left(0,\sigma_{c}^{2}\right)$$

where $\varepsilon_{c}$ is a standard normal random variable with relative standard deviation (RSD) $\sigma_{c}/\left[\hat{i}\right]$ chosen between 0.02 and 0.20. Note that the noise increases with the concentration level. The range of the RSDs was chosen based on the data from Széliová et al.,^[^ ^17^ ^]^ where median RSDs of the concentration measurements from three to six replicates were 1.7–7.1% (mean RSDs 5.3–19.8%). One thousand concentration profiles were generated for each metabolite. Afterwards, an equation of form (1) was fitted to each profile with R function nls, using $q_{i}$ and ${\left[i\right]}_{0}$ as the fitting parameters, thereby obtaining 1000 sets of exchange rates. Standard errors (SEs) of the fitted rates were used as a measure of accuracy. For a graphical representation of the workflow see Figure A1, Sim 1.

In the second version of the simulations, biomass concentrations were perturbed, in addition to concentration profiles. Cell concentration data BM was simulated with Equation (3),

(3) $$\text{BM}={\hat{\text{BM}}}_{0}\;e^{\hat{\mu }t}+\varepsilon_{B}\;\text{with}\;\varepsilon_{B}∼N\left(0,\sigma_{B}^{2}\right)$$

where t is time of cultivation (0–90 h), ${\hat{\text{BM}}}_{0}$ is the initial biomass (the same as in Equation (1)) and $\hat{\mu }$ is the growth rate from the reference FBA. Normally distributed noise of 2–20% ($\sigma_{B}/{\hat{\text{BM}}}_{0}\;e^{\hat{\mu }t}$ = 0.02–0.2) was added in the same way as described above for the concentration profiles. The parameters μ and ${\text{BM}}_{0}$ were estimated by fitting the simulated biomass concentrations with an exponential growth function using R function nls. Note that the estimated growth rate (μ) was used for the simulations of metabolite concentrations with Equation (1) (instead of the growth rate predicted by the reference FBA) (Figure A1, Sim 2).

### 2.3 FBAs with simulated exchange rates

The simulated exchange rates ($q_{i}$) and the standard errors (SEs) were used as constraints for FBA. The lower and upper bounds of the exchange reactions were set to $q_{i}$ ‐ SE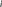 and $q_{i}$ + SE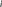, respectively. In rare cases when it was not possible to fit Equation (1) to the concentration profiles due to high noise, the exchange rates were left unconstrained. Biomass production was maximized.

## 3 RESULTS

First, we defined a reference state for further simulations. We used experimental data and GSMMs of 11 CHO cell lines/conditions^[^ ^17^ ^]^ in the exponential phase of a batch culture and performed FBA as described in Methods. Uptakes of cysteine and tryptophan were computationally estimated due to the lack of quantitative data. We maximized biomass production and obtained growth rates ($\hat{\mu }$) in the range 0.0263–0.046 h^‐1^. These were considered as “true” values for the subsequent simulations. Except for DXB11 models, the predicted uptake rates of cysteine and tryptophan exactly corresponded to the requirements for the biomass synthesis and were used for further simulations. (In the DXB11 models cysteine is not essential and the predicted cysteine uptakes were lower than the biomass requirement.)

### 3.1 Low exchange rates are highly inaccurate

**FIGURE 1:**
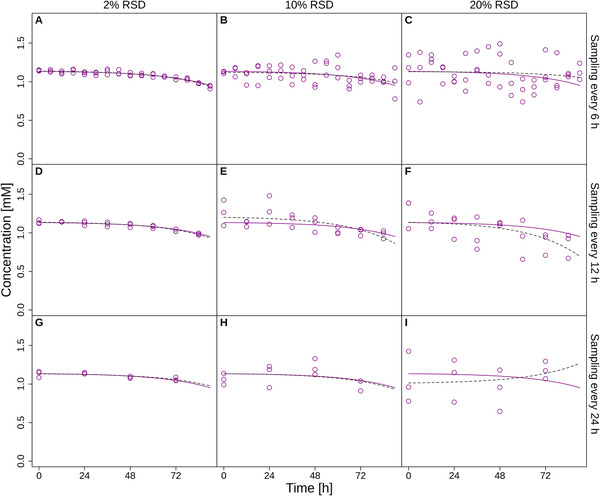
Simulated reference profiles (solid lines) and sampled perturbed concentrations (points) of histidine during the exponential phase of a batch cultivation at a sampling frequency of 6 h (panels A, B, C), 12 h (panels D, E, F) or 24 h (panels G, H, I) with 2% (panels A, D, G), 10% (panels B, E, H) or 20% (panels C, F, I) RSD of the replicate concentration measurements. Dashed lines represent fits to the data using Equation (1). Data are shown for cell line K1par‐8mMAP.

First, 1000 sets of concentration profiles of 23 extracellular metabolites (AAs, ammonium, glucose, and lactate) were simulated by adding normally distributed, independent random errors with a mean of zero and a RSD ranging from 2% to 20% to all reference distributions. Three replicates were simulated (representing three independent experiments) and the distributions were sampled at three regular intervals—every 6, 12, or 24 h (corresponding to 16, 8, or 4 time points throughout the batch, respectively). Figure 1 exemplarily shows simulated histidine concentration profiles for sampling intervals of 6, 12, and 24 h and RSDs of 2%, 10%, and 20%.

Next, we calculated exchange rates by fitting Equation (1) to the simulated concentration profiles and analytically evaluating its derivative. Low sampling frequency and high error can result in bad fits that in some cases may even predict secretion instead of consumption (Figure 1, panel I).

**FIGURE 2:**
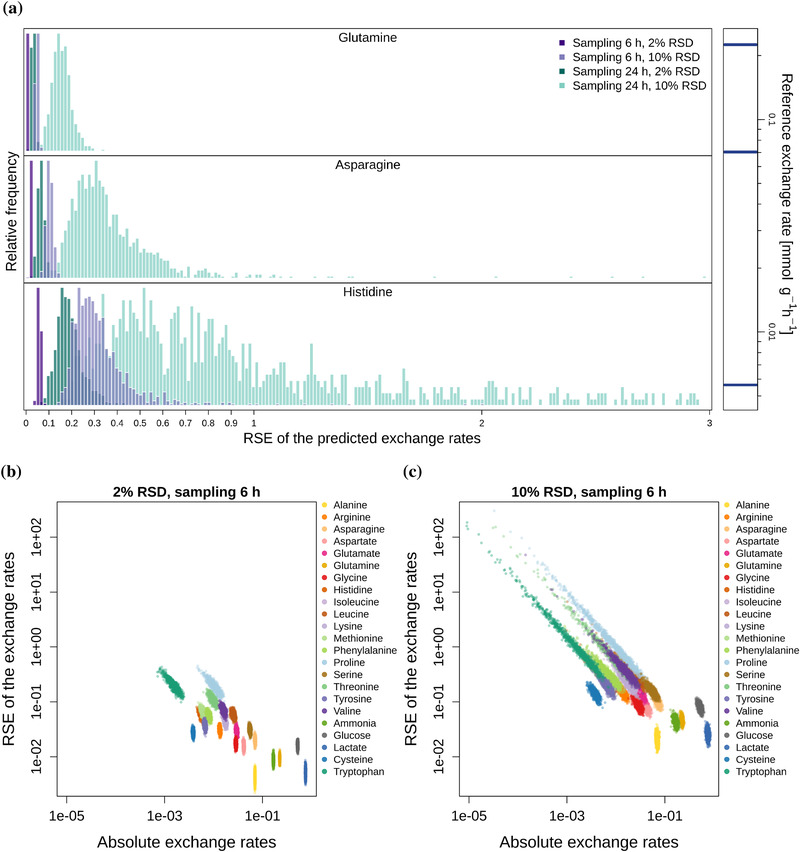
Panel a: distribution of the RSEs of three example uptake rates (for cell line K1par‐8mMAP). The bars on the right side indicate the magnitudes of the uptake rates (top, glutamine; middle, asparagine; bottom, histidine). Purple colors show the distributions for sampling frequency of every 6 h, green colors for frequency of every 24 h, each for 2% and 10% RSD of the concentration measurements. Bottom: RSEs of the exchange rates as a function of the absolute values of exchange rates shown for 2% (panel b) and 10% (panel c) RSD of the concentration data.

We used the SEs to assess the accuracy of the calculated exchange rates ($q_{i}$). Although the simulated concentration data has the same RSD for all metabolite concentrations, the relative standard errors (RSEs) of the calculated exchange rates (SEs divided by the absolute value of the exchange rates, $q_{i}$) markedly vary. More specifically, the closer the exchange rate is to zero, the higher the RSE and the wider the distribution (Figure 2, Figure A2). Figure 2a shows examples for a sampling frequency of 6 h (purple colors), where the simulated RSD of the concentrations is 2% and the medians of the RSEs of the rates are 1% for glutamine (high uptake), 2% for asparagine (medium uptake) and 6% for histidine (low uptake). 10% RSD of concentration measurements leads to median RSEs of the rates of 5%, 10%, and 29% for glutamine, asparagine, and histidine, respectively. If the sampling interval is increased from 6 to 24 h (green colors), the resulting RSEs of the exchange rates triplicate. For instance, with a 10% concentration error, the median RSE of the uptake of histidine increases up to 86% and the distribution has a very long tail. Figure A3 shows the relationship between RSDs of the metabolite concentrations and RSEs of the calculated exchange rates at different sampling frequencies for cell line K1par‐8mMCD.

### 3.2 Growth predictions are strongly sensitive to small metabolite concentration errors

**FIGURE 3:**
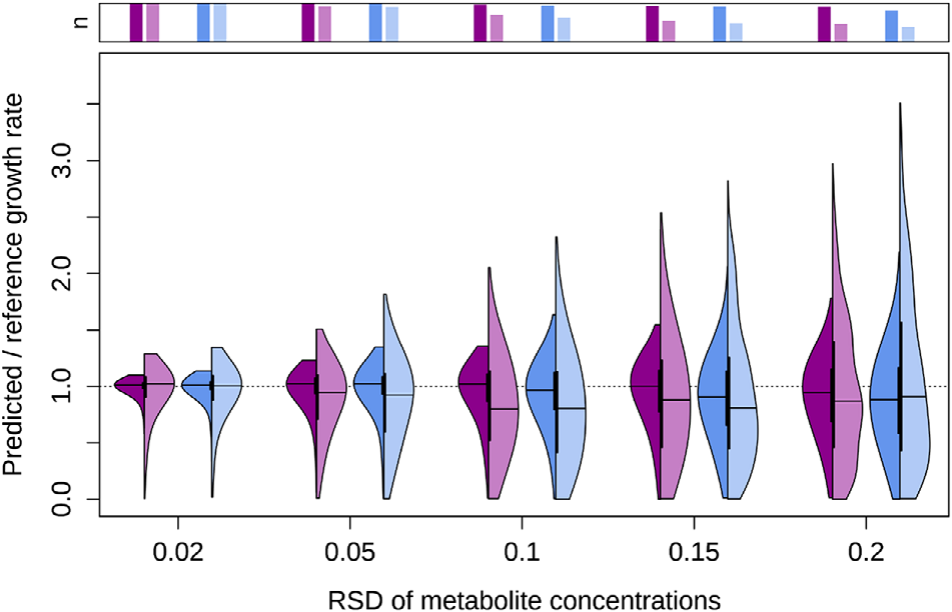
FBA predictions at different concentration RSDs for two cell lines (purple: K1par‐8mMAP, blue: HYher‐8mMCD). The left side of the violin plots corresponds to sampling every 6 h, the right side to every 24 h. The barplots above the plots indicate the fraction of feasible FBA solutions. The apparent cutoffs on the top at lower RSDs are artifacts due to the visualization.

We constrained the GSMM iCHO1766 with the computed exchange rates $\left[q_{i}-{\text{SE}}_{i},q_{i}+{\text{SE}}_{i}\right]$ and maximized growth with FBA. Figure 3 shows the ratios between predicted and reference growth rates for two selected cell lines in different media (K1par‐8mMAP and HYher‐8mMCD). The distribution of growth rates gets wider with the increasing RSD in the metabolite concentrations and decreasing sampling frequency. At RSD of 20% and sampling frequency of 24 h, the predicted growth rate varies between zero and three times the true value. The distribution gets skewed because the growth rates cannot be negative. Figure A4 displays the RSDs of the predicted growth rates as a function of concentration RSDs, showing that the data follows a square root law. That means that even small concentration errors lead to significant deviations of the predicted growth rate. However, growth rate RSD for concentration RSD of 20% is expected to be only twice higher than the one for concentration RSD of 5%.

With increasing RSD and decreasing sampling frequency we observe a growing number of infeasible FBA problems (indicated by the bars on top in Figure 3). At RSD of 20% and sampling frequency of 24 h, more than half of the associated linear problems are infeasible. The predictions for the slower cell line (HYher‐8mMCD, blue colors) are consistently worse, which can be explained by the on average lower uptake rates, which increases the RSEs of the calculated uptake rates (Figure A2).

Apart from the metabolite concentrations, the calculation of exchange rates takes the growth rate μ as an input (Equation (1)). To check the impact of the error of the cell concentration measurements on the calculations of the exchange rates and the FBA predictions, we added noise not only to the metabolite concentrations but also to the cell concentrations, where we introduced 6% RSD (the accuracy of Vi‐CELL XR (Beckman Coulter), an automated cell counting device commonly used to quantify cell concentration). Figure A5d shows that the predictions are practically indistinguishable from the results in Figure 3. Growth rates are typically high enough (0.02–0.04 h^‐1^)^[^ ^17^ ^]^ and thus can be determined with high accuracy. To verify this, we analyzed the variation in the estimated growth rates at 2–20% measurement RSDs (Figure A5a and A5b). Even if the measurement error is 20% and sampling only once per day, the RSDs calculated from 1000 estimated growth rates were below 15%. At the selected measurement RSD of 6%, the RSDs of the estimates were only 2–3%. Furthermore, we compared the RSDs of the estimated exchange rates with or without perturbing the cell concentration by 6%. Figure A5c shows the maximum observed differences between exchange rate RSDs estimated with or without perturbing the cell concentration. Based on these results, we concluded that measurement errors of the growth rate have only a small effect on our simulations and need not be considered further.

Another variable in Equation (1) is the cell dry mass (contained in ${\text{BM}}_{0}$). This parameter is commonly determined only once for each particular cell line and reused for all further experiments, so the error in this parameter was not considered and the value was fixed to the previously measured cell line/condition specific values.^[^ ^17^ ^]^

### 3.3 Essential amino acids determine the growth rate predictions

Next, we determined which exchange rates have the largest influence on growth rate predictions with FBA. Based on Figure A3, we hypothesized that the metabolites with largest relative error have the largest impact. Thus, if those were measured accurately, it would improve the prediction accuracy. To test this, we constrained the RSD of the concentrations to 2% for the top seven metabolites with highest RSEs of the rates. This strongly improves the prediction accuracy as measured by a decrease in the interquartile ranges for K1par‐8mMAP (before/after): 0.13/0.09, 0.25/0.12, 0.37/0.16, and 0.47/0.2 for 5–20% RSDs, respectively (Figure A6a). Note that for 2% RSD, the results are the same, because in both cases all metabolites have 2% RSD. The seven most error‐prone exchange rates were uptakes for proline, tryptophan, threonine, valine, methionine, leucine and histidine, the last 6 of which are essential components. The results for the cell line HYher‐8mMCD show a similar trend (Figure A7).

As the uptake of essential AAs cannot be compensated by the uptake of other AAs, we next tested whether growth rate is limited mainly by the uptake rates of essential AAs. Therefore, we split the exchange rates into two groups ‐ group 1: uptake rates of essential AAs (histidine, isoleucine, leucine, lysine, methionine, phenylalanine, threonine, tryptophan, valine, cysteine, arginine) and group 2: all other exchange rates. We varied the RSD of the concentration data for one group, while keeping the RSD of the second group constant. When the error of the essential AA concentrations is kept constant (group 1), varying the error of the nonessential AAs has no effect on the FBA predictions (Figure A6c and Figure A7c, left halves). On the other hand, increasing the error of the essential AA concentrations leads to a large increase in the variability of the FBA solutions (Figure A6c and Figure A7c, right halves) and also has a minor effect on the number of infeasible FBA problems.

The uptake rates and biological functions of the essential AAs vary. Some are used only for the generation of biomass (see next paragraph), while others are also metabolized for other purposes (e.g., generation of energy), so we expected that their impact on the FBA predictions might differ. Therefore, the next grouping was based on the biomass requirements of the essential AAs, which can be calculated as growth rate × AA coefficient in the biomass equation. Again, the exchange rates were divided into two groups: group 1: essential AAs with uptake rates smaller than 1.5× their biomass requirement (for cell line K1par‐8mMAP: arginine, lysine, phenylalanine, threonine, tryptophan, cysteine; for cell line HYher‐8mMCD: arginine, leucine, lysine, methionine, phenylalanine, tryptophan, cysteine); group 2: all other exchange rates. Figure A6d and Figure A7d show that varying the concentration errors of AAs in group 1 (right halves), which are used only for biomass formation, has a much bigger impact on FBA predictions than varying the errors of metabolites in group 2 (left halves). Conversely, it makes only a tiny difference whether or not we keep the error of group 2 at 2% or leave the exchanges unconstrained (right halves of Figures A6d and A7d vs. Figure A8). Finally, no such effects were apparent when we repeated the same procedure with random groupings (Figure A9).

**FIGURE 4:**
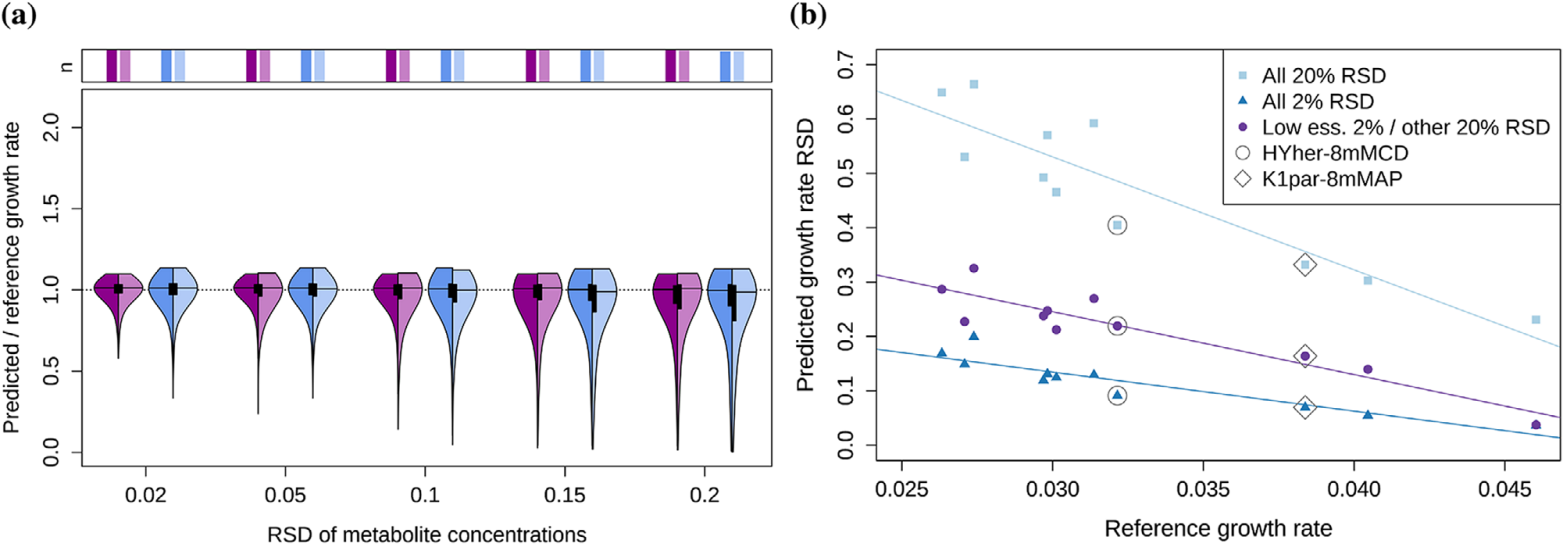
Panel a: the most important exchange rates for FBA predictions are cell line specific—the left halves show the predictions when the AAs with low normalized uptakes are chosen according to the cell line specific data; in the right halves they are chosen based on the data of the other cell line. In both cases, the RSDs of the chosen AAs are set to 2%, the remaining RSDs are varied. Purple: K1par‐8mMAP, blue: HYher‐8mMCD. The barplots above the plot indicate the fraction of feasible FBA solutions. The apparent cutoffs on the top are artifacts due to the visualization. Panel b: Prediction accuracy as a function of growth rate for three cases—the RSDs of metabolite concentrations were all set to 20% (”All 20% RSD”), 2% (”All 2% RSD”) or the low uptake AAs were set to 2% and the rest to 20% (”Low ess. 2%/other 20% RSD”). The lines represent linear fits. Data are shown for the sampling frequency of every 6 h.

Together these data demonstrate that the quality of FBA predicted growth rates is (primarily) determined by the uptake rates of essential AAs, which are used mostly for generation of biomass. The set of the relevant AAs is cell line‐specific (according to the interquartile ranges, Figure 4a). However, measuring them accurately leads to a larger improvement than accurate measurements of the high‐error AAs (Figures A6,A7). Note that only two out of six AAs for K1par‐8mMCD (threonine, tryptophan) and three out of seven for HYher‐8mMCD (leucine, methionine, tryptophan) from the ”low uptake” group are also in the ”top 7 high errors” group.

Because the most important AAs are cell line specific, we extended the analysis to 11 datasets from Széliová et al.^[^ ^17^ ^]^ which include nine different cell lines, some of them in various media compositions (CD‐CHO or ActiPro, 8 or 0 mM glutamine). We calculated normalized uptake rates for all datasets (for each specific biomass composition) and compared the lists of AAs with uptakes smaller than 1.5× of the biomass requirements. For all cell lines/conditions the list included lysine, phenylalanine and arginine, except for DXB11 datasets, where arginine was predicted to be nonessential (this is because no cell line specific model for DXB11 was available^[^ ^9^ ^]^ and arginine is not essential in the generic model). Isoleucine was not part of the low essential group in any of the datasets. For the remaining essential AAs, the normalized uptake rates varied among cell lines and conditions, but no consistent pattern was observed.

We ran FBAs for all the datasets, where we varied the RSDs of all metabolites (2–20% RSD) or kept the RSDs of low uptake essential AAs at 2% (and varied the rest). Figure 4b shows that in all cases measuring the low uptake AAs accurately improves predictions. No cell line or condition specific effect was observed. However, we found a dependence on growth rate—the lower the reference growth rate, the worse the quality of the predicted growth rates. This is likely due to the fact that slower cells also have lower uptake rates, which in turn have bigger relative errors (as shown in the previous section).

### 3.4 Accurate concentrations and high sampling frequency are needed for growth comparisons

Often, the goal of FBA is to compare (predicted) growth rates between different cell lines or conditions.^[^ ^16^, ^28^, ^29^ ^]^ Therefore, we wanted to determine how often we are able to correctly identify the growth rate difference between two selected cell lines at the different sampling frequencies or RSDs of the concentration measurements. Again the difference from the reference FBAs was regarded as a ”true” difference in the growth rates (0.0384 h^‐1^ for K1par‐8mMAP vs. 0.0321 h^‐1^ for HYher‐8mMCD, resulting in a 0.0063 h^‐1^ or 16% difference). As described earlier (Section 3.2), we ran 1000 FBAs for the two cell lines at fixed sampling frequencies and concentration RSDs (Figure 3). We compared the predicted growth rates of the two cell lines for all possible pairs ($10^{6}$ pairs for each sampling frequency and each RSD). In some cases the FBAs for one or both cell lines had no feasible solution, so the total number of comparisons was lower (Figure A10).

In Figure A10 we normalized the predicted differences by the expected difference in growth rate (0.0063 h^‐1^) and plotted them as cumulative distributions. As expected, with increasing RSDs of the concentration data and decreasing sampling frequency, there is a growing number of solutions that incorrectly identify the faster‐growing cell line and the distributions get broader. For example at a daily sampling frequency and 20% RSD (Figure A10, right plot, green line), there are around 20% cases where the predicted difference is 5× higher than the ”true” difference and almost 50% cases where the predicted difference is in the opposite direction.

**FIGURE 5:**
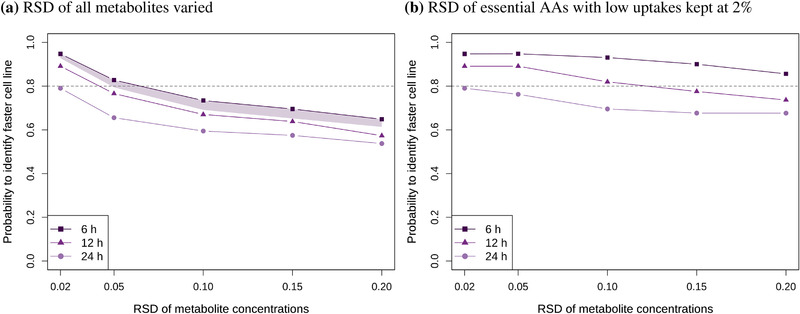
Percentage of FBAs where faster‐growing cell line is correctly identified at different concentration RSDs and sampling frequencies. In panel a, concentration RSDs of all metabolites are varied between 2% and 20%. In panel b, the RSDs of low uptake essential AAs are kept at 2% (as in Figures A6d and A7d), while the RSDs of the remaining metabolites are varied between 2% and 20%. The shaded area in panel a represents results with irregular sampling schedules with 4 samples per day (see Methods for details) or sampling every 12 h with six instead of three replicates.

Figure 5a shows the (cumulative) probability of correctly predicting the faster‐growing cell line. It can be used to determine the measurement accuracy and sampling frequency that are needed to identify the faster cell line in at least 80% of cases. If the concentration measurements are accurate enough, sampling frequencies of 6 h and 12 h lead to correct identification in at least 80% of cases. This probability quickly decreases with larger RSDs in the concentration measurements. In the worst case, less than 60% FBAs correctly predict which cell line grows faster, which is only slightly better than chance.

We also checked whether it is necessary that the time points are equally spaced, as sampling every 6 h is impractical to do in a laboratory. Therefore, we simulated data with different sampling schedules where the sampling time points are close together during the (working) day (every 4 or 2.5 h), followed by a longer interval without sampling (12 or 16.5 h). In addition, we simulated sampling every 12 h, but with six rather then three replicates. The results for these alternative sampling schedules lie within the shaded area in Figure 5a.

All previous simulations were done with three replicates at each time point (as illustrated in Figure 1). We wanted to test whether doubling the replicate number leads to a considerable improvement in the predictions of growth rate. Figure A11a shows the probability of identifying the faster‐growing cell line with six replicates. The improvement compared to three replicates is very small. For comparison, we increased the number of replicates to 100 (Figure A11b), although this is usually not feasible in a laboratory. The improvement is only modest, especially for the higher concentration RSDs. If the RSD is 15% or bigger and sampling is done on a daily frequency, there is still a more than 20% chance to incorrectly identify the faster‐growing cell line.

Finally, Figure 5b shows the probability of correctly predicting the faster‐growing cell line when the RSDs of essential AAs with low uptakes are kept at 2% and the RSDs of the remaining AAs are varied (as in Figure A6d and Figure A7d, left halves). For the highest sampling frequency (every 6 h), the faster cell line is identified in at least 80% cases for all RSDs.

**FIGURE 6:**
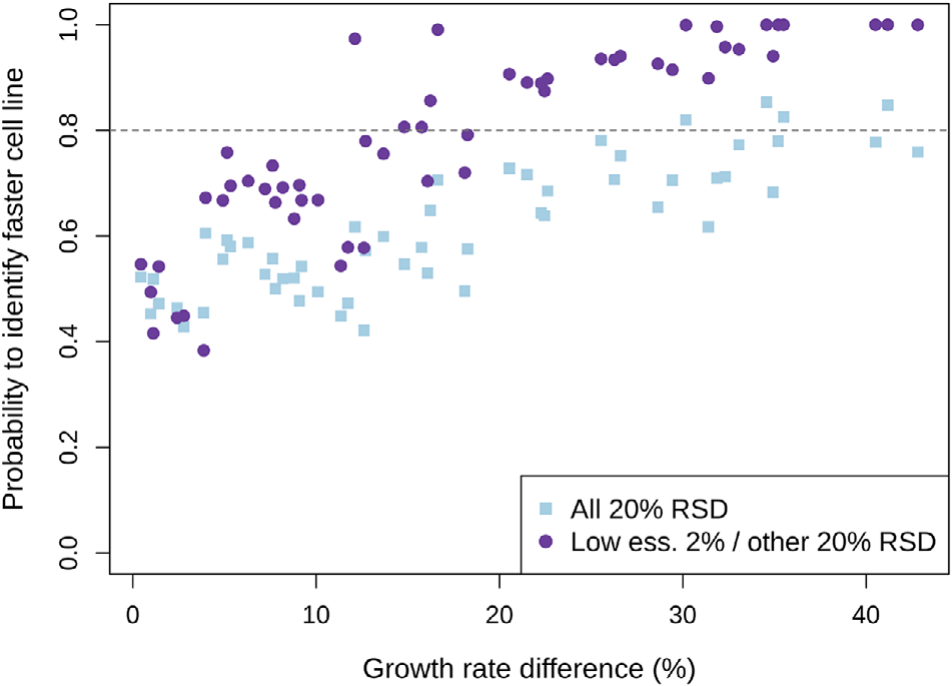
Probability to identify faster cell line as a function of growth rate difference at sampling frequency of every 6 h for two cases: ”All 20% RSD”—RSDs of concentration measurements of all metabolites are 20%; ”Low ess. 2%/other 20% RSD”—RSDs of low essential AA concentrations are 2%, the rest 20%.

In the previous paragraphs we showed comparisons between the two selected datasets with 16% difference in growth rate. However, the size of the difference also affects the prediction accuracy. We expected that the bigger the growth rate difference, the easier it is to detect it. To verify this, we compared all possible pairs of the 11 datasets,^[^ ^17^ ^]^ resulting in 55 comparisons, and plotted the probability to detect the faster cell line as a function of the growth rate difference. Two cases are shown as examples – 1. RSDs of all metabolite concentrations are 20% and 2. the low uptake AAs are measured accurately with 2% RSD and the rest with 20% RSD (Figure 6). Generally, the higher the growth rate difference, the easier it is to detect the faster cell line. However, for the case with 20% RSD of all the measurements, the difference cannot be reliably detected for most of the tested combinations, even at the biggest growth rate differences. Reducing the measurement error of the low uptake AAs leads to an improvement in the predictions and the faster cell line can be reliably detected if the difference is at least 20%. Small growth rate differences (0.4–10%) could not be reliably detected in any of the cases.

## 4 DISCUSSION

Systems level analysis of CHO cells is needed to design rational engineering and optimization strategies to streamline cell line and process development. The GSMM of CHO ^[^ ^9^ ^]^ provides a basis for constraint‐based metabolic modeling methods such as FBA. To obtain useful predictions, it is necessary to feed the model with accurate data, mainly exchange rates of extracellular metabolites. CHO cells are cultivated in complex media and consume or secrete numerous metabolites, including glucose, lactate, ammonium, and all AAs.

Previously, we showed that accurate quantification of exchange rates is essential for good predictions of CHO growth rates by FBA; to achieve the required accuracy it is necessary to analyze a sufficient number of time points throughout the relevant culture phases.^[^ ^17^ ^]^ However, we did not systematically analyze the effect of measurement error and sampling frequency on the rate calculations and growth rate predictions, nor did we identify which metabolites have the biggest impact. These points are addressed here.

In Figure 2, we showed that the smaller the exchange rate, the higher the relative error of the rate, which was already pointed out by Hädicke et al.^[^ ^30^ ^]^. The analysis also shows the importance of a sufficiently high sampling density throughout the culture. Figures 2 demonstrates that sampling only once per day (or four time points during the exponential phase, as is standard practice) leads to errors in the rates that are several times larger than the errors of the concentration data. There are also more extreme values which are basically unusable for any meaningful analysis with FBA. This is demonstrated in Figure 3 by the big variance and the high number of infeasible solutions. Consequently, insufficient measurement accuracy and sampling frequency also make it almost impossible to reliably compare the predicted growth rates of two cell lines (Figure 5).

More than 20 exchange rates are used as inputs for FBA and their impacts on the predictions of growth rates are vastly different. We expected that the prediction accuracy might improve if we measure those metabolites accurately that have the biggest relative errors of the rates. Although this approach led to a big improvement in the predictions, further improvement was observed when we considered the biological roles of the metabolites. All AAs are used as building blocks for the biomass, but there are big differences in their exchange rates and metabolism. Some can be synthesized in CHO, while others need to be taken up from the medium. Most of the essential AAs are consumed at very low rates and are predominantly used for the synthesis of the biomass but some can also be partially catabolized. We expected that the AAs which are solely used for the biomass synthesis would have the biggest impact on the growth rate predictions, which was confirmed by our analysis in Figures A6 and A7. Interestingly, the uptake rates of glucose and glutamine, which are the main energy sources for CHO,^[^ ^31^ ^]^ have no impact on the growth rate predictions, suggesting that energy provision is not the limiting factor for the predicted growth rates. However, this only applies to the cultures supplemented with an excess of energy sources, which was the case for all the batch cultivations in Széliová et al.^[^ ^17^ ^]^. If energy sources in the medium were insufficient, their uptake rates would likely become more important for predictions of growth rate.

In a similar analysis by Goudar et al.,^[^ ^32^ ^]^ error propagation from metabolite concentrations to rates and then to metabolic fluxes was analyzed and revealed that lesser metabolic fluxes (AA metabolism) were strongly influenced by the errors in the greater exchange rates (e.g., glucose, lactate), but not by the lesser exchange rates (AAs). In contrast, our analysis showed that low exchange rates of essential AAs had the biggest impact on the predictions of growth rate (which was not analyzed in Goudar et al.^[^ ^32^ ^]^). Among other differences between the studies is that in Goudar et al. a small metabolic network was used and not all relevant nutrient exchange rates were considered (e.g., all AAs). Furthermore, the experiments were done in perfusion cultures, where the procedure for determination of rates is different from batch cultivation, since they are in steady state. Nevertheless, both of these studies complement each other by showing the importance of the accurate rate determination for metabolic modeling.

Bayer et al.^[^ ^33^ ^]^ point out that the choice of the rate calculation method largely impacts the accuracy of the calculated rates. They concluded that fitting a cubic smoothing spline function is better than a step‐wise integration. As here we analyzed data from the exponential phase, an exponential function was more appropriate than a spline function. Similar to the spline function, an exponential function is fitted to the data from the whole process and therefore should not be influenced by experimental noise as much as step‐wise integration.

Our analysis showed that to obtain accurate FBA predictions of growth rates, essential AAs with low uptake rates should be quantified with the highest possible accuracy and at a sufficient sampling frequency. These aspects should be considered when planning experiments. The RSDs simulated here represent the overall variation among replicates. This can be divided into biological variation and the variation stemming from the measurement errors. Hädicke et al.^[^ ^30^ ^]^ show how the contributions of these errors to the total variance can be analyzed. Generally, the biological variance is bigger than the measurement variance and cannot be influenced, but it can be estimated from historical data, if available. On the other hand, measurement variance can be reduced by choosing an appropriate method that can accurately quantify the AAs that have the biggest impact on predictions and considering all other aspects that could impact the data quality (e.g., storage at ‐80 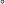
C immediately after sampling, avoiding freeze‐thawing). The measurement accuracy of six example AAs (important for K1par‐8mMAP) is discussed in the supplementary Section A1.

When planning experiments, simulations similar to the ones presented here can be performed to determine whether a certain accuracy and sampling frequency are sufficient to answer the research questions. Although the growth rate difference between two conditions/cell lines is not known beforehand, researchers can define what difference they want to be able to detect and adjust the experimental design accordingly. Realistically, growth rate differences below 15% are unlikely to be detected by FBA even if high effort goes into performing an experiment.

This work showed the effect of measurement error and sampling frequency on growth rate predictions and highlighted the difficulty of obtaining accurate uptake rates of the AAs with low uptakes even when the experimental errors are low. These results are specific to the use of biomass objective function. Schinn et al.^[^ ^34^ ^]^ reported that the objective function has a big impact on the prediction accuracy and the biomass objective function was correlated with poor predictions. A suggested alternative was minimization of cytosolic NADPH regeneration, which was correlated with good predictions. In another work, Chen et al.^[^ ^35^ ^]^ proposed using minimization of the uptake of a nonessential metabolite (e.g., glucose) as the objective function. The authors fix the growth rate to the experimental value and predict the uptake rates of essential AAs. As a result, the experimental error of these AAs would not affect the FBA solution.

## CONFLICT OF INTEREST

The authors declare no commercial or financial conflict of interest.

## AUTHOR CONTRIBUTIONS

Conceptualization: Diana Széliová, Dmytro Iurashev, David E. Ruckerbauer, Michael Melcher, Jürgen Zanghellini; Data curation: Diana Széliová; Formal analysis: Diana Széliová, Dmytro Iurashev, Michael Melcher; Investigation: Diana Széliová, Dmytro Iurashev; Methodology: Diana Széliová, Dmytro Iurashev, David E. Ruckerbauer, Gunda Koellensperger, Michael Melcher, Jürgen Zanghellini; Software: Diana Széliová; Validation: Diana Széliová; Visualization: Diana Széliová; Writing‐original draft: Diana Széliová, Dmytro Iurashev, David E. Ruckerbauer, Gunda Koellensperger, Michael Melcher, Jürgen Zanghellini; Writing‐review and editing: all; Funding acquisition: David E. Ruckerbauer, Nicole Borth, Jürgen Zanghellini, Project administration: David E. Ruckerbauer, Nicole Borth, Jürgen Zanghellini; Supervision: Nicole Borth, Michael Melcher, Jürgen Zanghellini; Resources: Gunda Koellensperger.

## Supporting information

Supporting Information

## Data Availability

Data are available at https://doi.org/10.17632/5vn5m33wpr.1.The data that support the findings of this study are openly available in Mendeley Data at https://doi.org/10.17632/5vn5m33wpr.1, reference number https://doi.org/10.17632/5vn5m33wpr.1
